# The relevance of pacing strategies in managing symptoms of post-COVID-19 syndrome

**DOI:** 10.1186/s12967-023-04229-w

**Published:** 2023-06-08

**Authors:** Alaa Ghali, Valentin Lacombe, Camille Ravaiau, Estelle Delattre, Maria Ghali, Geoffrey Urbanski, Christian Lavigne

**Affiliations:** 1 Department of Internal Medicine and Clinical Immunology, Angers University Hospital, 4 Rue Larrey, 49000 Angers, France; 2 Department of General Medicine, Faculty of Medicine of Angers, Angers, France

**Keywords:** Post-COVID syndrome, Myalgic encephalomyelitis/chronic fatigue syndrome, Pacing strategies, Adherence, Outcomes

## Abstract

**Background:**

Post-COVID-19 syndrome (PCS) shares many features with myalgic encephalomyelitis/chronic fatigue syndrome (ME/CFS). PCS represents a major health issue worldwide because it severely impacts patients’ work activities and their quality of life. In the absence of treatment for both conditions and given the beneficial effect of pacing strategies in ME/CFS, we conducted this study to assess the effectiveness of pacing in PCS patients.

**Methods:**

We retrospectively included patients meeting the World Health Organization definition of PCS who attended the Internal Medicine Department of Angers University Hospital, France between June 2020 and June 2022, and were followed up until December 2022. Pacing strategies were systematically proposed for all patients. Their medical records were reviewed and data related to baseline and follow-up assessments were collected. This included epidemiological characteristics, COVID-19 symptoms and associated conditions, fatigue features, perceived health status, employment activity, and the degree of pacing adherence assessed by the engagement in pacing subscale (EPS). Recovery was defined as the ability to return to work, and improvement was regarded as the reduction of the number and severity of symptoms.

**Results:**

A total of 86 patients were included and followed-up for a median time of 10 [6–13] months. Recovery and improvement rates were 33.7% and 23.3%, respectively. The EPS score was the only variable significantly associated with recovery on multivariate analysis (OR 40.43 [95% CI 6.22–262.6], p < 0.001). Patients who better adhered to pacing (high EPS scores) experienced significantly higher recovery and improvement rates (60–33.3% respectively) than those with low (5.5–5.5% respectively), or moderate (4.3–17.4% respectively) scores.

**Conclusion:**

Our findings demonstrated that pacing is effective in the management of patients with PCS, and that high levels of adherence to pacing are associated with better outcomes.

**Supplementary Information:**

The online version contains supplementary material available at 10.1186/s12967-023-04229-w.

## Background

According to the World Health Organization, post-COVID-19 syndrome (PCS) is defined as signs and symptoms that develop during or after an infection consistent with COVID-19, present for more than 12 weeks and are not attributable to alternative diagnoses. Symptoms may be new following initial recovery from an acute COVID-19 episode or persist from the initial illness [1]. They are heterogeneous and often involve multiple organ systems. Approximately 10–35% of COVID-19 non-hospitalized patients experience post-COVID symptoms [2–4].

Given the fact that fatigue was reported to occur after several viral and non-viral infections [5], it is unsurprising that it is one of the main symptoms that characterizes PCS. In a recent review of studies on PCS, 92.6% of participants from 55 studies presented with fatigue [6]. Other frequent symptoms that persist beyond 6 months after acute COVID-19 infection include post-exertional malaise (PEM), cognitive dysfunction, sleep disturbances, orthostatic intolerance, myalgia, headaches, dyspnea, palpitations, dizziness, and balance disorders [7].

PCS represents a major health issue worldwide because it severely impacts patients’ work activities and their quality of life [4]. A recent study showed that PCS was linked to unemployment and inversely associated with working full time [8].

PCS shares many features with myalgic encephalomyelitis/chronic fatigue syndrome (ME/CFS), which is often triggered by a variety of infectious agents, especially Epstein-Barr virus [9], and occurs predominantly in previously healthy and active females [10]. The majority of PCS symptoms are similar to those encountered in ME/CFS [11], and get worse or relapse after even minimal physical or mental exertion as in the case of ME/CFS [7]. Worsening of symptoms after a stressor that was normally tolerated before disease onset defines the PEM, which is the cardinal feature of ME/CFS [12]. PEM was found to persist in 73.3% of PCS patients beyond 6 months [7]. In the same way, some comorbidities, such as postural orthostatic tachycardia syndrome (PoTS) [13–16] and mast cell activation syndrome (MCAS) [17, 18], are commonly encountered in both conditions. The activation of mast cells could play a role in the hyper-inflammatory response to COVID-19 [19] and may give rise to similar symptoms of MCAS [20]. Owing to the overlap in the clinical features of PCS and ME/CFS, some authors have suggested that patients with PCS are likely to develop prolonged symptoms that meet ME/CFS criteria including PEM [21] and have proposed the term post-COVID-19 ME/CFS [7, 11, 22]. The exact pathophysiology of both conditions remains unclear, but some mechanisms including mitochondrial dysfunction, systemic and neuro-inflammation, and inappropriate immune response were reported in both PCS and ME/CFS patients [23].

In the absence of treatment for ME/CFS, preventing the exacerbation of the disease baseline symptoms and PEM occurrence constitutes the cornerstone of disease management. This is based on implementing pacing strategies that aim at coping with the decreased and inconsistent energy levels, which are constantly experienced by patients with ME/CFS [24]. These strategies, were used to manage some chronic medical conditions other than ME/CFS, such as multiple sclerosis [25], rheumatoid arthritis [26], and pain [27]. They consist of adapting and adjusting the different patients’ activities in terms of physical, cognitive and emotional effort within the limits imposed by the illness [28]. Pacing is very similar to the energy envelope theory [29] or “staying within the envelope”, which not only seeks to avoid overexertion responsible for baseline symptom exacerbation and PEM occurrence, but also underexertion [24, 29]. Consequently, perceived energy levels will increase over time and fatigue levels decrease, allowing patients to progressively perform higher levels of physical and cognitive activities [29]. Pacing activities according to available energy resources helps prevent worsening of the disease [28] and improves patients’ quality of life [30] while preserving adequate activity levels. The Center for Disease Control (CDC) recommends pacing for PCS patients experiencing PEM [31], and the National Institute for Health and Care Excellency (NICE) proposes self-management interventions for these patients [32].

However, to our knowledge, the relevance of pacing strategies in PCS patients has not yet been assessed. Based on our positive experience with pacing at our national referral center for ME/CFS patients, we conducted this study to assess the effectiveness of pacing strategies in managing the symptoms of patients with PCS, especially in terms of reducing fatigue levels and preventing PEM occurrence.

## Methods

This retrospective study was approved by the Ethics Committee of Angers University Hospital (2022/174) and was conducted in compliance with the Helsinki Agreement. Data collection was approved by the French Data Protection Authority (CNIL).

We retrospectively reviewed all medical records of patients diagnosed with PCS who attended the outpatient clinic of the Internal Medicine Department of Angers University Hospital, France between June 2020 and June 2022, and were followed up until December 2022. We included all patients who fulfilled the WHO definition of PCS [1]. We excluded patients who had medical records with missing or incomplete data, especially about pacing adherence and patients who were lost to follow-up.

The initial and different follow-up assessments of all patients were standardized and conducted by the same physician. Each assessment included taking a detailed medical history and a thorough physical examination. Patients were systematically asked about their employment activity, time at the workplace and travel to and from work, sick leave, and whether they returned to work or not.

At the initial assessment, all patients underwent an overall assessment of their epidemiological characteristics, the initial COVID-19 episode, and COVID 19-related symptoms and signs present for more than 12 weeks including, persistent, recurrent, and/or new-onset symptoms. Basal fatigue levels and its impact on patient activities, especially the current occupational status, were evaluated for all patients. In addition, special attention was paid for symptoms consistent with mast cell activation and those suggestive of autonomic dysfunction. At the end of the initial assessment, all patients were advised to apply pacing strategies, and were informed that the main objective of pacing is to prevent the exacerbation of symptoms, in particular fatigue and PEM occurrence, while remaining as active as possible. Pacing strategies rest on three pillars, which are as follows: 1. Staying within the limits of the energy envelope through identifying the current limits of the physical and mental functional capacity in different activities of daily living and not exceeding them, prioritizing activities, combining periods of activity with periods of rest, and splitting and switching activities. 2. Preventing worsening/relapse of symptoms and PEM occurrence. This requires identifying factors that trigger PEM such physical, mental and emotional stressors, orthostatic intolerance, hormonal factors in women, environmental factors (humidity and extreme temperatures), sensory stimuli (light, noise and smells), certain foods, infectious events, etc. In a number of patients, the onset of PEM is preceded by the appearance of new symptoms different from baseline manifestations such as mood disorders, nausea, headaches, vertigo, dyspnea, tingling or burning sensations, and others. These symptoms could be warning signals for PEM. Their identification could help preventing PEM occurrences or reducing their intensity [33]. 3. Cautious and progressive increase in the activities can be achieved only when symptoms are stabilized. Each patient received education in the form of a leaflet explaining the main bases of pacing and providing helpful hints for implementing pacing strategies. All patients were asked to keep a diary recording the current limits of their physical and mental functional capacity in different activities of daily living, factors that trigger fatigue or any other baseline symptoms, and possible warning signals for PEM (Additional file 1). Patients having symptoms consistent with mast cell activation, PoTS, neurocognitive impairment, or psychiatric disorders were referred to a specialist, if this was not already in place.

For all patients, follow-up assessments included an evaluation of self-reported health status, fatigue levels, persistent recurrent or new symptoms, the impact of the condition on patient’s activities, in particular on occupational status, and the degree of adherence to pacing strategies.

The health status of patients was subjectively assessed by asking them to describe changes in their current health state since the previous assessment: recovered = 5, significantly improved = 4, slightly improved = 3, stationary = 2 or worse = 1.

Fatigue was assessed in all patients by means of the Fatigue Severity Scale (FSS) [34]. This reliable and valid tool measures the impact of fatigue and detects change over time [35]. The FSS includes nine items rated on seven-point scales from 1 (completely disagree) to 7 (completely agree). A mean fatigue score that ranges from 1 to 7 was obtained by averaging the nine items. A mean FSS score ≥ 4 was indicative of clinically significant fatigue, and a reduction of 0.5 points was considered to be clinically significant [36].

The degree of adherence to pacing strategies was evaluated by means of the engagement in pacing subscale (EPS) of the Activity Pacing and Risk of Overactivity Questionnaire [37]. This subscale was previously used to measure reported engagement in pacing in multiple sclerosis patients [38]. All patients of the current study were asked to score each of the five items of the questionnaire on a scale of 1 to 5 (1 = never; 2 = rarely; 3 = sometimes; 4 = often; 5 = very often). A mean score that ranges from 1 to 5 was calculated by averaging the five items (Additional file 2). In the absence of a validated threshold that defines high levels of engagement in pacing, we set the cut-off score ≥ 4 defining high patient adherence to pacing. EPS scores between 3 and 3.9 corresponded to moderate pacing adherence, while scores < 3 corresponded to low adherence.

Recovery was defined as the complete remission of symptoms, and the capacity to resume pre-illness levels of physical, cognitive, and social functioning with no further need for pacing strategies. Recovered patients were able to return to work, on a full or part-time basis. Improvement was defined as the reduction in the number or severity of symptoms, and the capacity to achieve certain pre-illness levels of physical, cognitive, and social functioning while still needing pacing strategies. Improved patients were not able to return to work.

We classified the study population into 3 groups for comparative analysis: recovered patients (R group), improved patients, (I group), and patients who did not show any sign of recovery or improvement (no-R/I group).

Quantitative data were presented in medians and quartiles, and were compared between two groups for univariate analysis using a Student’s t-test or a Mann–Whitney test according to distribution normality, assessed by using the D’Agostino-Pearson test. Comparison of quantitative data between three groups was performed by using an ANOVA or a Kruskal–Wallis test according to the normality of distribution. Qualitative data were presented as absolute values and percentages, and were compared using the Fisher’s test or Chi-square test as appropriated. Multivariate analysis was performed by means of binary logistic regression on variables associated with recovery by comparing between R group and no-R/I group patients. The variables included in the model were age, sex, and those showing significant statistical difference between R and no-R/I groups in univariate analysis. The odds ratios (OR) were presented with a 95% confidence interval (CI). Time-to-event curves for cumulative incidence of improvement or recovery were presented as Kaplan–Meier curves, and compared with a log-rank test. Loss of follow-up was censored. The alpha risk was set at 5%. The analyses were performed using Graphpad Prism v6.01 (Graphpad Software, La Jolla, CA, USA) and Jamovi software v2.3.9.

## Results

**Fig. 1:**
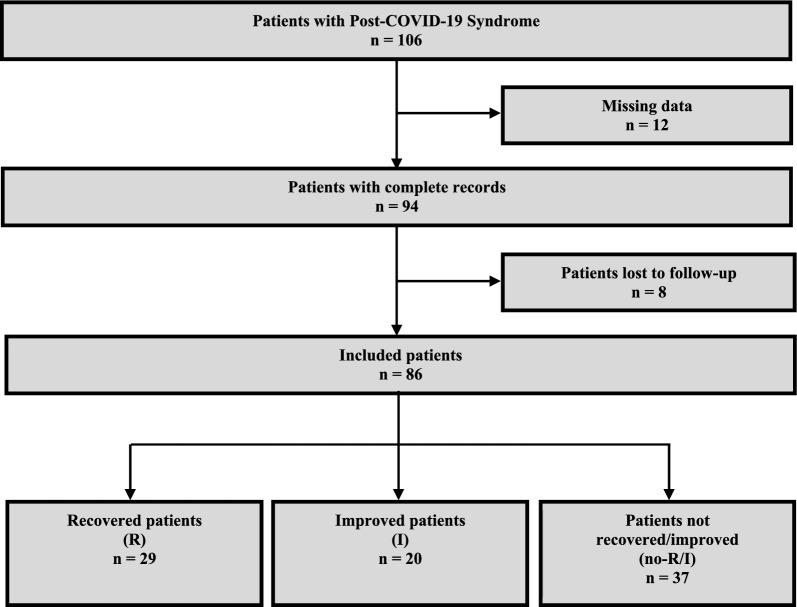
Flowchart

Among the 106 patients who fulfilled the inclusion criteria, 20 patients were excluded (12 with missing medical record data and 8 lost to follow-up). In total, 86 patients were included with a male to female ratio of 1:4.4. The median age at disease onset was 41 [33–48] years, and the median time of follow-up was 10 [6–13] months. Fatigue was the most frequent symptom, reported by 84/86 (97.7%) patients, followed by myalgia (53/86, 61.6%) and cognitive impairment (50/86, 58.1%). PEM was found in 32/86 (37.2%) patients (Additional file 3). The follow-up of patients showed that 29/86 (33.7%) experienced recovery and returned to work (R group), 20/86 (23.3%) showed improvement in their health status (I group), and 37/86 (43%) did not recover or improved (no-R/I group) (Fig. 1). In the R group, 10/29 (34.5%) patients returned to full-time employment, and the rest (19/29, 65.5%) returned to work on a part-time basis.

As summarized in Table 1, the comparison between the R group and the no-R/I group showed no significant statistical differences in univariate analysis except for the female sex (p = 0.002), the diagnostic delay (p = 0.047), the presence of cognitive impairment (p = 0.033), and the EPS score (p < 0.001). Both I and no-R/I groups were comparable for all variables except for the presence of a cough (p = 0.021), and the EPS score (p < 0.001).

**Table 1:** Comparison of recovered and improved patients with those who did not show recovery/improvement

	Patients’ groups			p-values	
	Recovered (R)	Improved (I)	Not recovered/improved (no-R/I)	R vs. no-R/I	I vs. no-R/I
Demographic characteristics					
Patients, n (%)	29 (33.7%)	20 (23.3%)	37 (43%)		
Female, n (%)	19 (65.5%)	16 (80%)	35 (94.6%)	**0.002**	0.086
Age at disease onset, years	41 [31–50]	41 [32–45]	41 [34–49]	0.529	0.428
Diagnostic delay, months	9 [6–13]	11 [6–17]	15 [9–18]	**0.047**	0.420
Time of follow-up, months	9 [6–12]	11 [6–14]	9 [6–12]	0.601	0.275
Clinical manifestations, n (%)					
Fatigue	29 (100%)	20 (100%)	36 (97.3%)	0.372	0.458
Post-exertional malaise	8 (27.6%)	7 (35%)	17 (45.9%)	0.126	0.424
Fever	2 (6.9%)	1 (5%)	4 (10.8%)	0.687	0.647
Chills/ sweats /flushing	10 (34.5%)	12 (60%)	16 (43.2%)	0.469	0.227
Thromboembolic disorders	0 (0%)	1 (5%)	3 (8.1%)	0.249	> 0.99
Dyspnea	15 (51.7%)	8 (40%)	23 (62.2%)	0.394	0.108
Cough	5 (17.2%)	1 (5%)	12 (32.4%)	0.256	**0.021**
Chest tightness/pain	7 (24.1%)	4 (20%)	6 (16.2%)	0.421	0.728
Cognitive impairment	12 (41.4%)	13 (65%)	25 (67.6%)	**0.033**	0.844
Brain fog	9 (31%)	14 (70%)	19 (51.4%)	0.097	0.173
Headaches/brain pressure sensation	12 (41.4%)	14 (70%)	18 (48.6%)	0.556	0.121
Neurosensory disturbances	1 (3.4%)	0 (0%)	4 (10.8%)	0.374	0.286
Vertigo/dizziness/balance problems	6 (20.7%)	8 (40%)	13 (35.1%)	0.198	0.716
Sleep disorders	11 (37.9%)	12 (60%)	16 (43.2%)	0.663	0.227
Sore throat	8 (27.6%)	2 (10%)	7 (18.9%)	0.404	0.470
Dysphonia	0 (0%)	1 (5%)	0 (0%)	> 0.99	0.350
Dysphagia	1 (3.4%)	1 (5%)	1 (2.7%)	> 0.99	> 0.99
Rhinorrhea	5 (17.2%)	1 (5%)	4 (10.8%)	0.490	0.647
Anosmia	5 (17.2%)	4 (20%)	4 (10.8%)	0.490	0.431
Ageusia	5 (17.2%)	4 (20%)	2 (5.4%)	0.490	0.169
Myalgia	18 (62.1%)	13 (65%)	22 (59.5%)	0.639	0.681
Arthralgia	5 (17.2%)	6 (30%)	9 (24.3%)	0.555	0.642
Numbness/tingling	2 (6.9%)	2 (10%)	3 (8.1%)	> 0.99	> 0.99
Mood disorders	11 (37.9%)	8 (40%)	16 (43.2%)	0.801	0.812
Gastrointestinal disorders	5 (17.2%)	5 (25%)	8 (21.6%)	0.656	0.771
Palpitation	10 (34.5%)	9 (45%)	19 (51.4%)	0.140	0.647
Conjunctivitis	1 (3.4%)	0 (0%)	1 (2.7%)	> 0.99	> 0.99
Associated conditions, n (%)					
Myalgic encephalomyelitis	8 (27.6%)	7 (35%)	17 (45.9%)	0.126	0.424
Postural orthostatic tachycardia syndrome	3 (10.3%)	6 (30%)	5 (13.5%)	> 0.99	0.358
Mast cell activation	10 (34.5%)	10 (50%)	16 (43.2%)	0.469	0.624
Baseline fatigue assessment					
Baseline FSS^a^ score	6.8 [6.3–7]	6.8 [6.6–7]	7 [6.8–7]	0.109	0.234
Degree of adherence to pacing					
Engagement in pacing subscale score	4.4 [4–4.8]	4.1 [4–4.4]	3 [2.8–3.2]	** < 0.001**	** < 0.001**

Qualitative data were expressed as absolute number and percentage, and compared between two groups by the Fisher’s exact test or the Chi-squared test, as appropriated. Quantitative data were expressed as median and quartiles and compared between two groups by the Student’s t-test or Mann–Whitney test according to the normality of distribution, assessed by using the D’Agostino-Pearson test

^a^FSS: Fatigue severity scale

The multivariate binomial logistic regression analysis showed that the EPS score was the only variable associated with recovery (OR 40.43 [95% CI 6.22–262.64], p < 0.001) (Table 2).

**Table 2:** Multivariate analysis of variables associated with recovery in R and no-R/I groups

	OR [95% CI]^a^	p-value
Female sex	0.12 [95% CI 0.01–1.54]	0.103
Age at disease onset	0.95 [95% CI 0.87–1.04]	0.276
Diagnostic delay	0.96 [95% CI 0.84–1.10]	0.52
Cognitive impairment	0.22 [95% CI 0.03–1.55]	0.128
Engagement in pacing subscale score	40.43 [95% CI 6.22–262.64]	** < 0.001**

Multivariate analysis was performed with binary logistic regression on variables associated with recovery by comparing between R group and no-R/I group patients. The variable to explain was recovery. The variables included were age at disease onset, sex, and those showing significant statistical difference between recovered patients and those who did not recover/improve in univariate analysis (p < 0.05)

^a^Odds Ratio with 95% Confidence interval

**Fig. 2:**
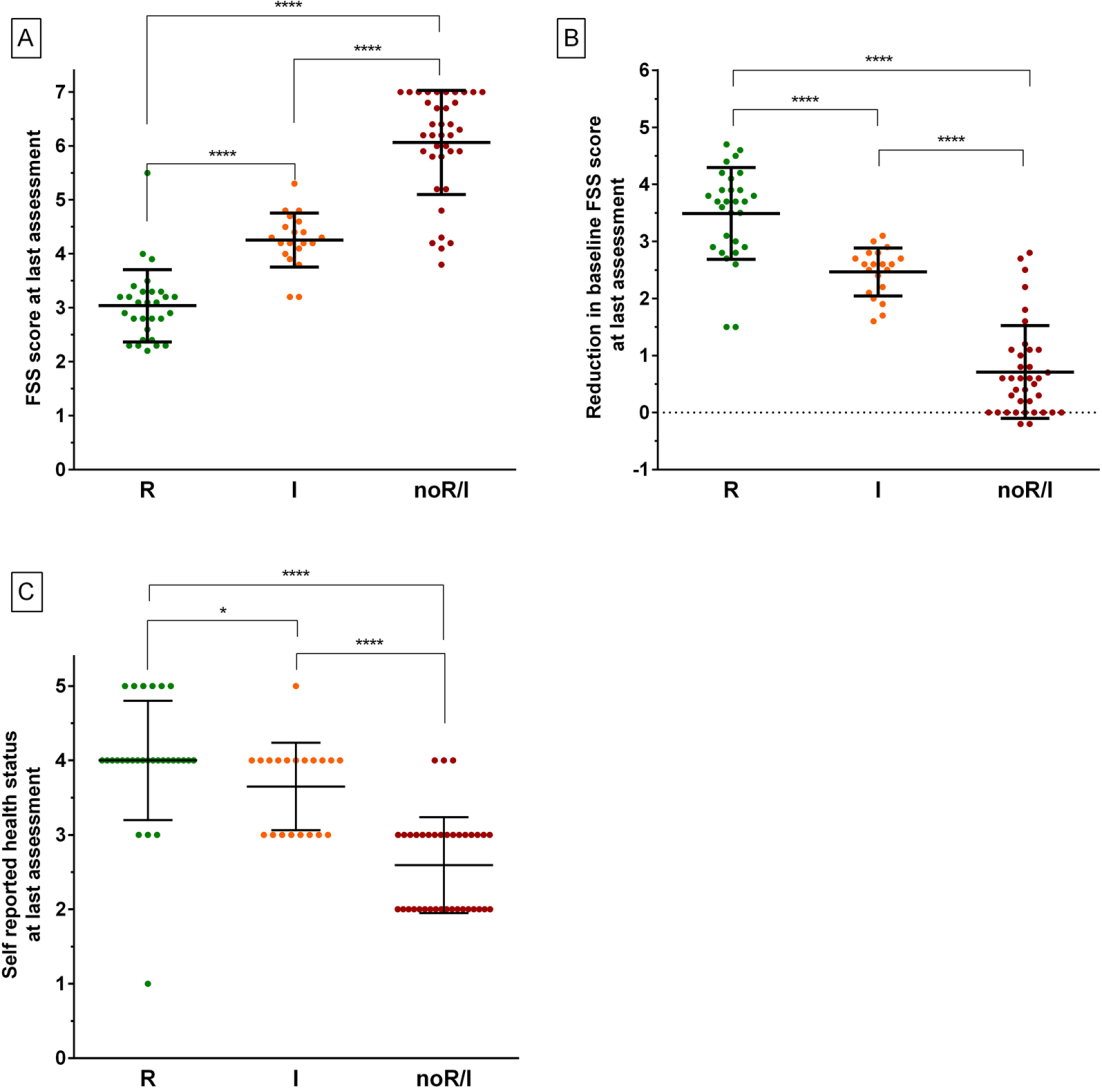
Comparison of the 3 groups of patients at last assessment in terms of FSS scores (**A**), reduction in FSS scores (**B**), and health status scores (**C**). *R* recovered patients, *I* improved patients, *no-R/I* patients not recovered/improved; *: p < 0.05; ****: p < 0.0001

In each of the 3 groups, the median FSS scores significantly decreased between baseline and last assessments (R group 6.8 [6.3–7] vs. 3.1 [2.6–3.3], p < 0,001; I group 6.8 [6.6–7] vs. 4.3 [4.1–4.5], p < 0,001; no-R/I group 7 [6.8–7] vs. 6.2 [5.8–7], p < 0,001). However, the median FSS scores at last assessment were significantly higher in the no-R/I group compared to the R and I groups, and differed significantly between the 3 groups (R group 3.1 [2.6–3.3], I group 4.3 [4.1–4.5], and no-R/I group 6.2 [5.8–7], p < 0,001, Fig. 2A). The reduction in the median baseline FSS scores at the last assessment was significantly higher in the R group (3.7 [2.9–3.9]) compared to the I group (2.6 [2.2–2.7], p < 0.001) and the no-R/I group (0.6 [0–1.1], p < 0.001). The reduction of median baseline FSS scores was also significantly different between the I group and the no-R/I group (p < 0.001, Fig. 2B). Similarly, the self-reported health status scores at last assessment were significantly higher in the R group (4 [4], p < 0.001) and I group (4 [3, 4], p < 0.001) compared to the no-R/I group (3 [2, 3], Fig. 2C).

**Fig. 3:**
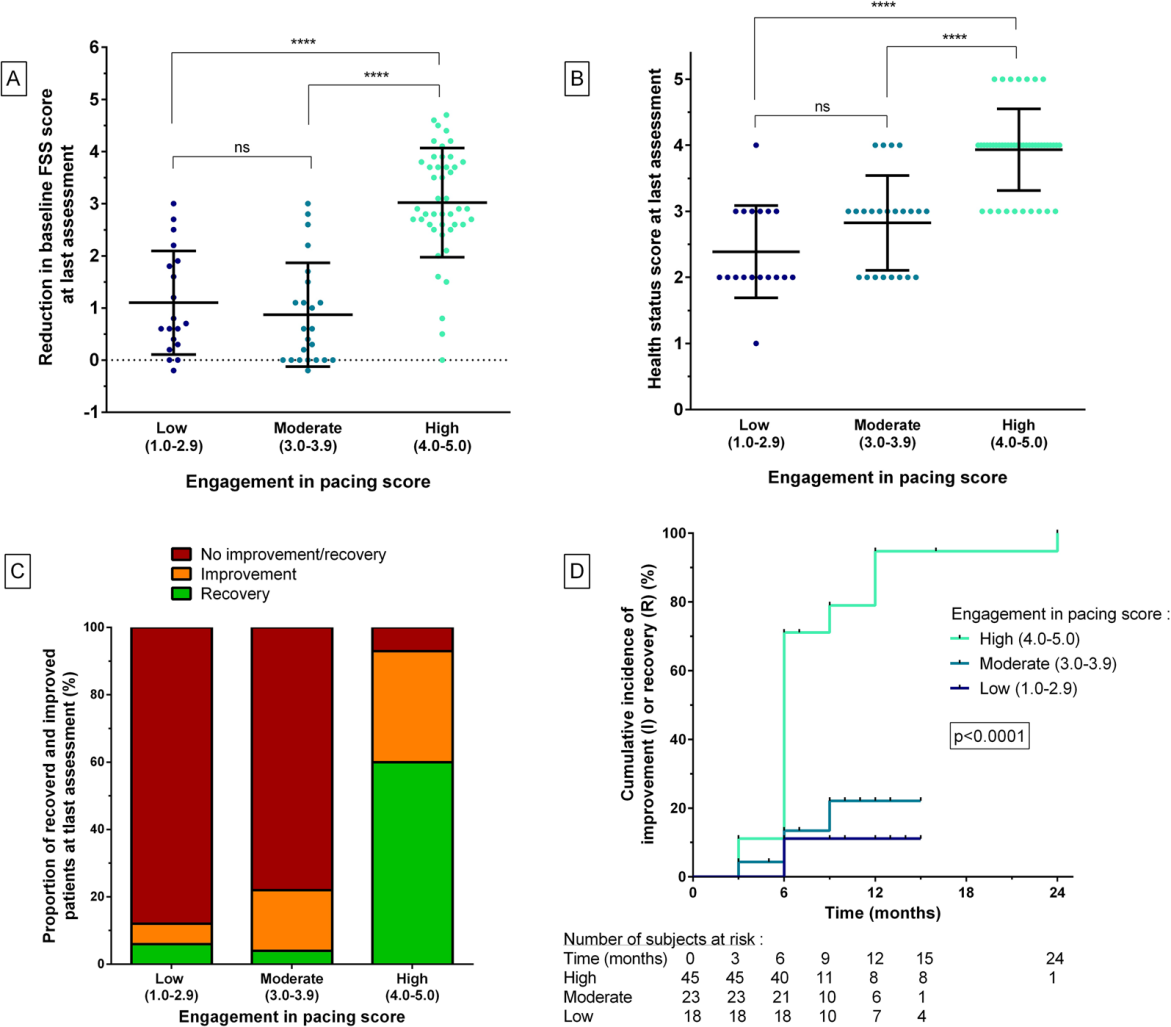
Evolution of patients according to the engagement in pacing subscale scores. **A**. Comparison of the reduction of the baseline FSS scores according to the engagement in pacing subscale scores. **B**. Comparison of the health status scores at the last visit according to the engagement in pacing subscale scores. **C**. Distribution of recovered, improved and not recovered/improved patients according to the engagement in pacing scores. **D**. Cumulative incidence of global improvement among patients who experienced recovery/improvement according to the engagement in pacing subscale scores (Log-rank test). *R* recovered patients, *I* improved patients, *no-R/I* patients not recovered/improved; ****: p < 0.0001

Compared to moderate (3.1–4.0) and low (< 3.0) EPS scores, high scores (≥ 4) were significantly associated with a higher reduction in FSS scores (p < 0.0001 for both comparisons) and with higher self-reported health status scores (p < 0.0001 for both comparisons). Moderate and low EPS scores did not significantly differ in terms of reduction in FSS scores or self-reported health status scores (Fig. 3A, B).

Moreover, high EPS scores were significantly associated with higher recovery/improvement rates compared to moderate and low scores: a recovery was observed in 27/45 (60.0%) patients with high scores, versus 1/23 (4.3%) and 1/18 (5.5%) in patients with moderate and low scores, respectively (p < 0.0001 for both comparisons). Furthermore, improvement was observed in 15/45 (33.3%) patients with high scores, versus 4/23 (17.4%) and 1/18 (5.5%) in patients with moderate and low scores, respectively (p < 0.0001 for both comparisons, Fig. 3C). Lastly, patients with high EPS scores improved more quickly than those with moderate and low scores (p < 0.0001, Fig. 3D).

Three months after the initial assessment and the implementation of pacing strategies, an improvement was observed in 6/49 (12.2%) patients, 5/6 (83.3%) of whom had high EPS scores. At 6-month follow-up, an improvement was observed in 32/49 (65.3%) patients, 29/32 (90.6%) of whom had high EPS scores.

## Discussion

Post-COVID-19 syndrome is a common condition that affects approximately 10–35% of COVID-19 non-hospitalized patients [2–4], and represents a major health issue worldwide because it severely impacts patients’ work activities and their quality of life [4]. Among the wide variety of PCS symptoms, fatigue is reported to be the most frequent complaint of PCS patients that persists beyond 6 months [7]. The majority of PCS symptoms are similar to those encountered in ME/CFS, especially the PEM [11], and get worse or relapse after even minimal physical or mental exertion [7]. PCS shares many other similarities with ME/CFS such as certain associated conditions, an unclear pathophysiology, an absence of biomarkers, and lack of an approved treatment. Pacing strategies are effective in improving the quality of life of ME/CFS patients and help prevent worsening of the disease [28, 30]. Pacing is used in the management of many other chronic conditions including autoimmune and neurological disorders. Pacing based on energy conservation, which is similar to the strategies used in ME/CFS, was successfully used in patients with RA [26] and those with MS [39]. Energy conservation includes alternating activity and rest, prioritizing activities, delegating tasks and using assistive devices to achieve everyday activities. Activity pacing is another form of pacing, which is more frequently used in chronic pain conditions and refers to the operant theory [40]. The operant theory-based interventions aim at achieving predetermined activity goals and gradual increasing of activity levels. Often, patient’s daily activities are divided into smaller, more manageable parts to avoid worsening of symptoms while maintaining a progressive increase in activity. Tailored activity pacing interventions appeared to be effective on symptoms of patients with MS [25] and knee and hip osteoarthritis [27] in terms of fatigue, physical activity, and pain. Similarly, the pacing-based exercise practice was reported to be relevant in children, and people with mental health and learning difficulties [41]. The principle of activity pacing differs from that used in ME/CFS and may be counter-productive in some cases. To our knowledge, the effectiveness of pacing in PCS was not reported before. Based on the beneficial effect of pacing strategies in preventing PEM occurrence and reducing fatigue levels in patients with ME/CFS, we conducted this study to assess the effectiveness of pacing in managing the symptoms of patients with PCS.

The study included 86 PCS patients with the median age at disease onset was 41 [33–48] years, and median time of follow-up was 10 [6–13] months. The higher prevalence of women (81.4%) in the current study was comparable to that previously reported [6, 42]. Fatigue was the most frequent symptom reported by 97.7% of patients, which is in line with the prevalence rate of 92.6% observed in a recent scooping review [6]. The prevalence of cognitive impairment in our study (58.1%) was also comparable to that reported (58.4%) by an international online survey. However, the number of patients who experienced PEM in the current study (37.2%) was much lower (73.3%) [7].

We observed a relatively high recovery/improvement rate (49/86 patients, 57%) after pacing implementation. About a third of patients (29/86, 33.7%) experienced recovery and returned to work, 10 (34.5%) of whom were able to return to full-time work and the rest returned to work on a part-time basis. Twenty (23.3%) patients showed improvement in their health status with a reduction in the number and severity of their symptoms enabling them to achieve certain levels of everyday activities using pacing.

The comparison between the R and no-R/I groups showed that, despite the fact that they were different on univariate analysis in terms of female sex, diagnostic delay, presence of cognitive impairment, and EPS score, the only variable that was significantly associated with recovery on multivariate analysis was the EPS score (OR 40.43 [95% CI 6.22–262.6], p < 0.001).

In order to assess the effectiveness of pacing strategies in the management of PCS patients, we compared the groups of recovered, improved, and non-recovered/improved patients in terms of fatigue levels, fatigue improvement, and perceived heath status. Fatigue levels were measured by means of the FSS, fatigue improvement was evaluated by the reduction in the baseline FSS scores at the last assessment, and the perceived health status by using the self-reported health status questionnaire. Included patients had severe baseline fatigue levels as shown by the high baseline FSS scores with no statistical difference between the 3 groups of patients (Table1). As expected, the greatest improvement in fatigue levels and the overall health status was observed in recovered patients who were thus able to return to work and to their normal performance prior to the COVID infection. Improved patients showed more improvement in fatigue levels and perceived health status compared to the non-recovered/improved patients, meaning this group only regained some of their pre-illness independence. These findings are consistent with the definition we gave for recovery and improvement and confirm the strength of our evaluation criteria.

In order to appreciate whether the degree of pacing adherence has an impact on fatigue levels, fatigue improvement, perceived heath status, and recovery rate, we used the 5-item engagement in pacing subscale [38]. We demonstrated that patients who adhered more closely to pacing were experiencing a higher degree of improvement in their fatigue levels and reported better perceived health status compared to those with moderate or low adherence. Consequently, these patients had higher recovery and improvement rates, and improved more rapidly than those with moderate or low adherence to pacing. While it is difficult to determine whether improvement of patients with low pacing adherence was spontaneous or due to low levels of pacing, results of the current study demonstrated that high adherence to pacing has led to higher and faster recovery rates.

On the other hand, it is important to mention that pacing is not an avoidance behavior, but a series of strategies that aim to prevent both overexertion and underexertion. In other words, pacing has the objective of maintaining the different activities within the limits of the available energy reserve. To attain this goal, pacing strategies require continuous adaptation and adjustment of the provided effort according to perceived energy levels. As a result of these strategies, energy levels will gradually increase with a reduction in the perceived fatigue, thus allowing patients to progressively conduct higher levels of physical and cognitive activities [29] and to improve their quality of life [30]. If not, pacing will at least prevent worsening of symptoms and disease evolution [28]. Recently, the CDC advised PCS patients experiencing PEM to apply pacing methods and to follow the same recommendations given for ME/SFC patients [31]. In addition, the NICE recommends a self-management support and interventions [32].

The main question that arises is how does pacing produce its beneficial effects in PCS and in ME/CFS. One possible explanation may be the imbalance in the cytokine activities. It is now known that patients with PCS [43–45], like those with ME/CFS [23, 46, 47], have elevated pro-inflammatory cytokine levels, mainly the interleukin-6 (IL-6). This one is an important pro-inflammatory cytokine involved in the development of fatigue in both autoimmune and non-autoimmune diseases [48]. In the context of exercise, however, IL-6 rises progressively for a short period and activates anti-inflammatory cytokines before its rapid drop in the post-exercise period [49]. The resultant anti-inflammatory cytokines induce a more prolonged anti-inflammatory effect and could be one explanation for the beneficial effect of exercise in healthy adults and the symptomatic improvement of some patients with chronic inflammatory disease, such as rheumatoid arthritis [50]. On the contrary, the elevated levels of pro-inflammatory cytokines including IL-6 in athletes with overtraining syndrome (OTS) may increase the production of reactive oxygen species (ROS) with a resultant imbalance in the redox state of the muscle, thus leading to impaired exercise performance. ROS can further elevate pro-inflammatory cytokines resulting in chronic inflammation responsible for the systemic manifestations of OTS including chronic physical and cognitive fatigue, sleep disorders, myalgia, arthralgia, and mood disorders [51, 52]. In this case, exercise will perpetuate the chronic inflammation by further increasing pro-inflammatory cytokines and the oxidative stress. It is therefore of interest to note that PCS and ME/CFS share a large resemblance with OTS, including the large number of clinical manifestations, chronic systemic inflammation, inappropriate immune response, mitochondrial dysfunction, absence of effective pharmacological treatment, deleterious effect of exercise, and beneficial effect of pacing.

For instance, a previous study on patients with CFS demonstrated that pro-inflammatory cytokines levels failed to decrease 48 h after moderate exercise, and concluded that the severity of post-exercise symptom exacerbation in severely fatigued patients was linked to cytokine activity [46]. Similarly, a recent qualitative study [53] conducted on 48 post-COVID patients showed that conventional rehabilitation programs, including graded-exercise therapy and respiratory rehabilitation, were not suitable for managing fatigue and PEM in these patients and need to be individualized. Moreover, unadapted return to pre-COVID physical activity levels such as everyday activity, work, or exercise, was often associated with worsening of symptoms. It is likely that pacing strategies through avoiding overexertion help to gradually restore pro- and anti-inflammatory cytokines balance, and thus improving the overall health status of patients.

A further possible explanation for the beneficial role of pacing in PCS and ME/CFS could be related to the mitochondrial dysfunction, which is known to be linked to fatigue [54]. Both conditions involve redox imbalance, impaired production of adenosine triphosphate (ATP), and high levels of oxidative stress [23]. Metabolic impairment of monocytes [55] and alteration of mitokine secretion [56] were also reported in PCS. Mitochondrial dysfunction results in an early switch of cells from aerobic to anaerobic pathways in response to exercise with the production of more lactic acid and 18 times less ATP per glucose molecule [57]. Adherence to pacing strategies maintain exercise level below the individual anaerobic threshold and thus probably avoid anaerobic glycolysis and lactate accumulation contributing to symptoms and signs of PEM.

One criticism of pacing is that it may cause deconditioning, and some authors have speculated that deleterious sequelae of exercise in a group of CFS patients were due to or maintained by physical and cardiovascular deconditioning. They concluded that physical reconditioning by means of a graded exercise program help improve the physical function of these patients [58, 59]. Nevertheless, a large number of studies demonstrated that deconditioning does not perpetuate or explain the symptoms in these patients [60, 61]. It has also been shown that graded exercise therapy (GET) and cognitive behavior therapy (CBT) are ineffective and may lead to the worsening of the disease and the occurrence of serious relapses [62–65]. For that reason, GET is no longer recommended for ME/CFS management and it was recently removed from the NICE guidance [66]. In this study, patients who adhered more strictly to pacing recovered and returned to their normal performance levels prior to the COVID infection, especially in terms of occupational functioning.

## Limitations and strengths

One source of weakness in this study was its retrospective character. However, all included patients were diagnosed, evaluated, and followed up by the same physician and underwent the same standardized procedure at the initial and follow-up assessments. Future prospective studies are required to confirm findings of the current work. Another limitation is the use of self-reported questionnaires to assess pacing and global health status, which could be a potential bias due to the subjectivity of these measures. However, in addition to these subjective measures, we set returning to work as an obligatory criterion for defining recovery, and used it to objectively assess the effect of pacing adherence on fatigue levels and health status perception. In this respect, we encourage researchers to adopt the employment status as an objective tool for assessing the evolution of subjective symptoms, in particular the fatigue. Lastly, making distinctions between the concepts of recovery and improvement in assessing pacing relevance could give more weight to our findings.

## Conclusion

To the best of our knowledge, our study is the first to assess the relevance of pacing in the management of patients with post-COVID syndrome. We observed a high recovery and improvement rates among these patients after the implementation of pacing strategies. We also demonstrated that high adherence to pacing strategies was effective for improving the overall health status, thus enabling patients to restore pre-illness performances and to return to work. The more the patient adheres to pacing, the higher the rate and the degree of recovery, and the more rapid the improvement. These findings emphasize the relevance of pacing in the management of PCS as is the case for ME/CFS. They further highlight the similarity between both conditions, and represent a strong argument for extending the use of pacing to PCS patients. To do this, it is important to raise awareness among primary care physicians about the substantial role of pacing in PCS management and establish educational programs to teach patients how to apply pacing strategies.

## Supplementary Information

**Additional file 1: Table S1**. Characteristics of the study population.

**Additional file 2: Figure S1**. Pacing Strategies for Managing Post-COVID-19 Syndrome.

**Additional file 3: Figure S2**. Assessment of Pacing Adherence in Patients with Post-COVID-19 Syndrome.

## Data Availability

The datasets used and/or analyzed during the current study are available from the corresponding author on reasonable request.

## Abbreviations

PCS: Post-COVID-19 syndrome
ME/CFS: Myalgic encephalomyelitis/chronic fatigue syndrome
WHO: World Health Organization
EPS: Engagement in pacing subscale
PEM: Post-exertional malaise
PoTS: Postural orthostatic tachycardia syndrome
MCAS: Mast cell activation syndrome
CDC: Center for Disease Control
NICE: National Institute for Health and Care Excellency
CNIL: French Data Protection Authority
FSS: Fatigue Severity Scale
R: Recovered patients
I: Improved patients
no-R/I: Patients not recovered/improved
OTS: Overtraining syndrome
ROS: Reactive oxygen species
ATP: Adenosine triphosphate
GET: Graded exercise therapy
CBT: Cognitive behavior therapy
