# Supplementary material for: The relevance of pacing strategies in managing symptoms of post-COVID-19 syndrome
Source: J Transl Med. 2023 Jun 8;21:375. doi: 10.1186/s12967-023-04229-w (PMC10248991; doi:10.1186/s12967-023-04229-w)
Supplement: Supplementary file 1 — Additional file 1: Table S1. Characteristics of the study population. [file 12967_2023_4229_MOESM1_ESM.doc]

**Table S1 Characteristics of the study population**

| **Demographic characteristics** |  |
| --- | --- |
| Patients, n | 86 |
| Female, n (%) | 70 (81.4) |
| Age at disease onset, years | 41 [33-48] |
| Diagnostic delay, months | 12 [7-17] |
| Time of follow-up, months | 10 [6-13] |
| **Clinical manifestations, n (%)** |  |
| Fatigue | 85 (98.8) |
| Post-exertional malaise | 32 (37.2) |
| Fever | 7 (8.1) |
| Chills/ sweats /flushing | 38 (44.2) |
| Thromboembolic disorders | 4 (4.7) |
| Dyspnea | 46 (53.5) |
| Cough | 18 (20.9) |
| Chest tightness/pain | 17 (19.8) |
| Cognitive impairment | 50 (58.1) |
| Brain fog | 42 (48.8) |
| Headaches/brain pressure sensation | 44 (51.2) |
| Neurosensory disturbances | 5 (5.8) |
| Vertigo/dizziness/balance problems | 27 (31.4) |
| Sleep disorders | 39 (45.3) |
| Sore throat | 17 19.8) |
| Dysphonia | 1 (1.2) |
| Dysphagia | 3 (3.5) |
| Rhinorrhea | 10 (11.7) |
| Anosmia | 13 (15.1) |
| Ageusia | 11 (12.8) |
| Myalgia | 53 (61.6) |
| Arthralgia | 20 (23.3) |
| Numbness/tingling | 7 (8.1) |
| Mood disorders | 35 (40.7) |
| Gastrointestinal disorders | 18 (20.9) |
| Palpitation | 38 (44.2) |
| Conjunctivitis | 2 (23.3) |
| **Associated conditions, n (%)** |  |
| Myalgic encephalomyelitis | 32 (37.2) |
| Postural orthostatic tachycardia syndrome | 14 (16.3) |
| Mast cell activation | 36 (41.9) |
| **Baseline assessment** |  |
| Fatigue severity scale score | 7 [6.7-7] |
| **Last follow-up assessment** |  |
| Fatigue severity scale score | 4.3 [3.2-6.2] |
| Engagement in pacing subscale score | 4 [3-4.4] |
| Self-reported health status score | 3 [3-4] |

Qualitative data were expressed as absolute numbers and percentages.

Quantitative data were expressed as medians and quartiles.
