# Supplementary material for: The relevance of pacing strategies in managing symptoms of post-COVID-19 syndrome
Source: J Transl Med. 2023 Jun 8;21:375. doi: 10.1186/s12967-023-04229-w (PMC10248991; doi:10.1186/s12967-023-04229-w)
Supplement: Supplementary file 2 — Additional file 2: Figure S1. Pacing Strategies for Managing Post-COVID-19 Syndrome. [file 12967_2023_4229_MOESM2_ESM.docx]

**Figure S1. Pacing Strategies for Managing Post-COVID-19 Syndrome**

Internal medicine department – Angers University Hospital

**What is “PACING”?**

Patients with chronic disease have the challenge of adapting themselves to their illness and coping with it to make their daily lives better.

- Pacing is defined as an energy management strategy aiming at maintaining one’s activities within the available energy envelope.
- PACING helps improving wellness of patients suffering from a chronic disease.

**Why “PACING” in post-COVID syndrome (PCS)?**

-PACING has undeniable benefits in the management of patients with myalgic encephalomyelitis (ME/CFS).

-PCS shares many characteristics with ME/CFS:

- PCS as most cases of ME/CFS occur after a viral infection.
- The symptoms of both conditions are comparable and their worsening/relapse occurs after exposure to physical, mental or emotional exertion.
- The post-exertional malaise (PEM) that characterizes the ME/CFS is encountered in a large number of patients with PCS.
- The pathophysiological mechanisms of PCS, even though they are not fully elucidated, resemble those of ME/CFS, especially an inappropriate immune response and a mitochondrial dysfunction.

-Mitochondria are intracellular organelles responsible for providing cells with the energy they need to ensure their adequate functioning. Their dysfunction leads to a defect in the production and storage of energy responsible for a reduction in the available envelope energy. So, limits of the energy envelope are rapidly reached leading to worsening/relapse of symptoms.

-A significant number of patients with PCS experience a state of severe physical, mental and emotional exhaustion after going beyond their functional capacities. This state of exhaustion, encountered in the ME/CFS and known as PEM, requires a long recovery period, sometimes lasting several weeks.

-Recurrent exacerbations of symptoms increases the severity of the symptoms, prevents a favorable evolution of the disease and negatively impacts patients’ quality of life.

-For this reason, identifying and respecting energy limits in patients with PCS is of great importance. Patients who develop post-COVID myalgic encephalomyelitis need to recognize the triggers and the warning signals of PEM to prevent its occurrence.

**What are the “PACING” strategies?**

**1-Stay within the limits of the energy envelope:**

**1.1 Identify the limits of your energy envelope**

- Evaluate the limits of your physical and mental functional capacity in different activities of daily living and note the thresholds in an activity diary.
- Remember that energy limits vary from day to day and even within the same day.

**1.2 Do not exceed your energy envelope**

- In order to improve your level of wellness, you should significantly reduce your activity for helping your body to move towards recovery. For instance, some people limit the time or distance they walk, the time they spend on the computer or phone, the time they spend with friends, or the time they spend in the household.
- How do you track your activity level?

There are several ways to track activity level. Some prefer hours and minutes; other may choose using the concept of marbles. For example, if you have 20 marbles available for use in a day, estimate the number of marbles needed for each activity e.g. 3 marbles for shower, 2 marbles for breakfast, 2 marbles for 20 minutes of reading emails, etc. In this way, you can decide to continue or not certain activities.

**1.3 Prioritize your activities**

- If your energy limits do not allow you to realize all the tasks you have to do, you have to prioritize your activities by starting with that of high level of importance.

**1.4 Plan breaks**

- Combining periods of activity with periods of rest can relieve your symptoms and allow you to gain more stability
- Note that resting means lying down with your eyes closed in a quiet place. Avoid watching TV, reading or talking on the phone while resting because these activities will consume energy. Relaxation methods or listening to soft music while resting are encouraged.
- The rest time and the number of breaks per day vary from person to person.

**1.5 Split activities**

- If you are able to perform an activity for 30 minutes, try to do it over two 15 minute-periods separated by a break. In this way, you will feel more productive and less symptomatic.

**1.6 Switch activities**

- Try to change the type of the activity; for instance, switch between physical, mental and social activities.
- Rest periods can be inserted between each change of activity.

**2- Prevent worsening/relapse of symptoms and PEM**

**2.1 Identify factors that trigger PEM**

Besides physical and mental stressors, there are other factors that can trigger MPE. Their identification is important to avoid worsening/relapse of symptoms and MPE onset. Examples:

- Positive or negative emotional load
- Orthostatic intolerance (long-standing position)
- Hormonal factors in women (premenstrual week – menstruation – pregnancy)
- Environmental factors (humidity – extreme temperatures)
- Stimuli (light – noise – smells)
- Certain foods
- Infectious events, etc.

These factors will considerably decrease your already reduced energy level. Therefore, it is important to avoid these stressors by implementing specific preventive measures. In case of unavoidable factors, further reduction of provided efforts will be required.

**2.2 Identify the warning signals of the MPE**

In a number of patients, the onset of MPE is preceded by the appearance of new symptoms different from those usually associated with ME/CFS. These symptoms could be warning signals for PEM. Their identification could help preventing PEM occurrences or reducing their intensity leading to improving disease prognosis.

The most common symptoms are:

- Mood disorders,
- Nausea,
- Headaches,
- Vertigo,
- Shortness of breath,
- Tingling or burning sensations, and others

**3- How to increase activity?**

The 3 “only” to be applied:

**3.1- Stabilize before increasing**

- Only when your symptoms have been improving steadily for at least 3 weeks, you can try to increase your activity level.

**3.2- Choose one activity at a time**

- Only one activity can be increased by week in the absence of worsening/relapse of symptoms.

**3.3- Gradually increase your activity**

- Increase your activity level by only 10%. A slow increase is ideal. Your symptoms are your guide.

**What do you expect from pacing?**

- Pacing is not a cure or a miracle solution, but a strategy that aims at coping with the decreased and inconsistent energy levels, which are constantly experienced by patients with long-lasting illness such as ME/CFS and PCS.
- Pacing consists in adapting and adjusting the different patients’ activities in terms of physical, cognitive and emotional effort within the limits imposed by the illness. This will permit increasing perceived energy levels over time, reducing fatigue levels, improving severity of other symptoms and allowing patients to conduct higher levels of physical and cognitive activities.

*********************
