# Supplementary material for: The relevance of pacing strategies in managing symptoms of post-COVID-19 syndrome
Source: J Transl Med. 2023 Jun 8;21:375. doi: 10.1186/s12967-023-04229-w (PMC10248991; doi:10.1186/s12967-023-04229-w)
Supplement: Supplementary file 3 — Additional file 3: Figure S2. Assessment of Pacing Adherence in Patients with Post-COVID-19 Syndrome. [file 12967_2023_4229_MOESM3_ESM.docx]

Figure S2: Assessment of Pacing Adherence in Patients with Post-COVID-19 Syndrome

**Angers University hospital - internal medicine department**

**Patient: name …………………….. first name …………………….. date of birth …. /…. /……..**

**assessment date: …. /…. /……..**

**Engagement in Pacing Questionnaire^1^**

Instructions: Circle the number that applies to you

|  | Never | Rarely | Sometimes | Often | Very often |
| --- | --- | --- | --- | --- | --- |
| 1. During the day I plan several moments to recover. | 1 | 2 | 3 | 4 | 5 |
| 2. I perform my activities at a slow pace. | 1 | 2 | 3 | 4 | 5 |
| 3. When performing my activities, I take my fatigue into account. | 1 | 2 | 3 | 4 | 5 |
| 4. I alternate intensive activities with less intensive activities. | 1 | 2 | 3 | 4 | 5 |
| 5. I divide my activities over the day. | 1 | 2 | 3 | 4 | 5 |

Total score =

Mean score =
